# Variability of pulmonary nodule volumetry on coronary CT angiograms

**DOI:** 10.1097/MD.0000000000030332

**Published:** 2022-09-02

**Authors:** Erique Pinto, Diana Penha, Bruno Hochhegger, Colin Monaghan, Edson Marchiori, Luís Taborda-Barata, Klaus Irion

**Affiliations:** a Universidade da Beira Interior Faculdade de Ciências da Saúde, Covilha, Portugal; b Imaging Department, Liverpool Heart and Chest Hospital NHS Foundation Trust, Liverpool, United Kingdom; c Pontificia Universidade Catolica do Rio Grande do Sul, Porto Alegre, Brazil; d Radiology Department, Liverpool Heart and Chest Hospital NHS Foundation Trust, Liverpool, United Kingdom; e Universidade Federal do Rio de Janeiro Faculdade de Medicina, Rio DE Janeiro, RJ, Brazil; f Universidade Federal Fluminense Faculdade de Medicina, Niteroi, RJ, Brazil; g Universidade da Beira Interior, Covilhã, Portugal; h Imaging Department, Manchester University NHS Foundation Trust, Manchester, United Kingdom.

**Keywords:** coronary CT angiogram, lung cancer, pulmonary nodule, incidental finding, volumetry, volume doubling time

## Abstract

This study aims to investigate the variability of pulmonary nodule (PN) volumetry on multiphase coronary CT angiograms (CCTA).

Two radiologists reviewed 5973 CCTA scans in this cross-sectional study to detect incidental solid noncalcified PNs measuring between 5 and 8 mm. Each radiologist measured the nodules’ diameters and volume, in systole and diastole, using 2 commercially available software packages to analyze PNs.

Bland-Altman analysis was applied between different observers, software packages, and cardiac phases. Bland-Altman subanalysis for the systolic and diastolic datasets were also performed.

A total of 195 PNs were detected within the inclusion criteria and measured in systole and diastole. Bland-Altman analysis was used to test the variability of volumetry between cardiac phases ([−47.0%; 52.3%]), software packages ([−50.2%; 68.2%]), and observers ([−14.5%; 27.8%]). The inter-observer variability of the systolic and diastolic subsets was [−13.6%; 31.4%] and [−13.9%; 19.7%], respectively.

Using diastolic volume measurements, the variability of PN volumetry on CCTA scans is similar to the reported variability of volumetry on low-dose CT scans. Therefore, growth estimation of PNs on CCTA scans could be feasible.

## 1. Introduction

Most scientific societies now recognize Coronary CT angiogram (CCTA) as a first-line examination in diagnosing and clinically managing stable coronary artery disease (CAD).^[1,2]^ CCTA has replaced invasive coronary angiography in diagnosing CAD and improved clinical outcomes for these patients.^[3–8]^ However, the increasing number of CCTA scans performed led to an increase in reported incidental extracardiac findings, with pulmonary nodules (PNs) being the most common (up to 28% of scans).^[9–11]^

The Fleischner Society recommends low-dose chest CT (LD-CT) follow-up of incidental solid, noncalcified PNs measuring between 5 and 8mm since the growth rate is a better marker for malignancy than size, calcifications, and morphological characteristics.^[12–14]^ As such, incidental PNs detected in CCTA scans and between 5 and 8 mm in diameter should have an LD-CT scan as soon as possible.^[12]^ We use dedicated volumetry tools to calculate the volume doubling time (VDT) and estimate the growth rate. When the VDT is shorter than 30 or longer than 400 days, the PN is more likely related to benign pathology (inflammatory causes or hamartomas, respectively). In comparison, a VDT between 30 and 400 days is suspicious for malignancy.^[15]^ However, these tools suffer from numerous known limiting factors, including the specific software package, nodule’s size, contact to other structures, use of contrast agent, slice thickness, slice overlap, “kernel,” degree of chest expansion, motion artifacts, and many others.^[15–20]^ In addition, factors related to cardiac function have also been suggested but not conclusively studied.^[21]^

**Figure 1.**
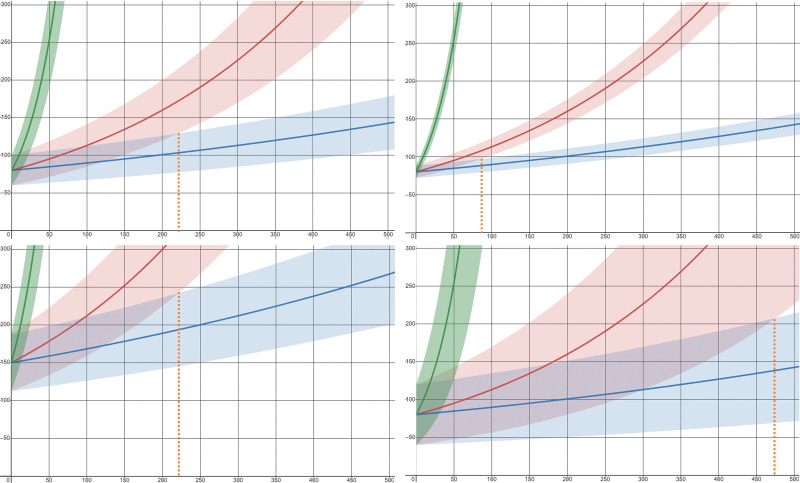
The importance of measurement variability in growth estimation. All graphs present expected volume measurements of 3 pulmonary nodules with different growth-rates [i.e., VDT = 30 days, as in inflammatory changes (green); VDT = 250 days, as in malignancy (red); and VDT = 600 days, as in benign pathology (blue)]. The shaded areas represent possible volume measurements over time given the different measurement variability values [left (top and bottom)—25%; top right—10%, and bottom right—50%]. Volume measurements in overlapping shaded areas cannot be confidently attributed to 1 growth rate or another. The starting volume of a nodule [top left—80 cc^3^; bottom left—150 cc^3^] does not change the optimal waiting time between follow-up scans for a confident discrimination between suspicious and benign pathology (dashed orange line), but the measurement variability can significantly shorten (top right) or extend it (bottom right).

Some authors have suggested that CCTA could be useful in lung cancer screening, given the considerable overlap of risk factors with CAD.^[22]^ Compared to LD-CT, the higher temporal resolution and electrocardiogram (ECG)-gating makes CCTA scans more robust to motion and breathing artifacts. Likewise, estimating growth from previous CCTA scans could expedite the diagnosis and decision-making while allowing for better resource management in radiology departments. However, growth estimation by PN volumetry is related to the measurement variability, as it impacts the optimal waiting time between follow-up scans.^[23]^ As such, a higher variability increases the overlap between benign and malignant pathology, reducing the discriminating power of volumetry (Fig. 1). The reported inter-scan variability of PN volumetry on LD-CT is around 25%, meaning that an increase in volume above 25% has a 95% likelihood of actual growth.^[24,25]^ Recently, Bartlett et al published their results on nonmetastatic PNs (28–150 mm^3^) and proposed that a threshold of 15% of volume increase could be more appropriate and that closer surveillance may be justifiable.^[26]^

Current guidelines and recommendations regarding the optimal waiting time between follow-up scans assume this variability of 25% for volumetry tools on LD-CT.^[23]^ For PN volumetry to apply to CCTA scans, its variability must be within these limits; otherwise, the current recommended waiting times would not be optimal. We found no published reports related to the variability of PN volumetry on ECG-gated CT scans.

This study aims to investigate the variability of PN volumetry on CCTA scans when applied to incidental solid PNs between 5 and 8 mm in diameter.

## 2. Methods

The Institutional Research Committee Review Board approved this retrospective cross-sectional study (observational, analytical) and waived the requirement for written informed consent due to the use of existing clinical data.

### 2.1. Study sample

The study sample includes consecutive patients who underwent a CCTA scan between January 1, 2016, and December 31, 2019, in our institution. All patients had one or more CCTA scans performed on the same equipment (Somatom Definition Flash; Siemens). Images were acquired during inspiration and contrast injection (75–95 mL of Niopam 370, at 5−7 mL/s), with anatomical coverage from the carina through the cardiac apex. Acquisition parameters include peak kilovoltage (kVp) between 100−120 kV; current modulation (CareDose 4D) with 320 mAs as reference; average acquisition time of 1−2 seconds; collimation of 128 × 0.6 mm; and pitch of 3.4. Reconstruction parameters include 2 mm and 0.75 mm overlapping slices through the prescribed range, with a B20f algorithm and field of view (FOV) of 22 cm.

The study’s inclusion criteria comprise scans with one or more incidental solid, noncalcified PN within the FOV in systole and diastole, measuring between 5 and 8 mm in long-axis diameter. Exclusion criteria comprise the absence of systolic (30–40%) or diastolic phase (70–80% of the cardiac cycle) in the archive; or when one of the cardiac phases did not present the PN in its FOV.

### 2.2. Observers and measurements

Two cardiothoracic radiologists with 10 (observer 1) and 5 years of experience (observer 2) identified nodules fitting the eligibility criteria within the study sample. In addition, a consensus decision between both observers and a third chest radiologist (with >25 years of experience) resolved disagreements regarding whether an appearance met the inclusion criteria.

Each observer measured each nodule using 2 different PN volumetry software packages in systole and diastole. The software packages used were Carestream Vue PACS v 11.4.01.1011 (Carestream Health, Inc, Rochester, NY) and Syngo via VB20 (Siemens Healthineers AG, Erlangen, Germany), as tool 1 and tool2, respectively.

Both tools performed semiautomatic segmentation by placing 1 seed point in the middle of the nodule. The observers did not correct the resulting segmentation. Still, they recorded the adequacy of the segmentation as “inadequate” if 3 consecutive segmentation attempts had failed or when the segmentation did not adequately represent the nodule. Also recorded were the observer’s initials; software package used; cardiac phase; long-axis diameter of the nodule; location of the nodule in axial and coronal planes; and nodule volume calculated using the volumetry tool.

### 2.3. Statistical analysis

The data were analyzed using SPSS software (ver. 26.0; IBM Corporation).

Descriptive statistical analysis of the included nodules was performed.

All cases with inadequate nodule segmentation were excluded from further analysis.

The association of volume measurements was tested using Kendall tau correlation coefficients (τ) between different cardiac phases, software packages and observers.

The Bland-Altman analysis is a well-known statistical tool for method comparison and has been used previously to study the variability of PN volumetry.^[27]^ Volume measurement agreement was tested by Bland-Altman analysis and plots. In addition, further inter-observer analyses of the systolic and diastolic data subsets were performed.

The estimation of the limits of agreement (LOA) in the Bland-Altman analysis used the nonparametric quartile analysis after the exclusion of normality of the differences of measurements. Possible reasons for this non-normal distribution include the presence of a natural limit (i.e., volume must be a positive number) and the restriction of the nodules’ diameter between 5 and 8mm (i.e., sorted by diameter).

## 3. Results

Of a total of 5973 candidate CCTA scans performed between 2016 and 2019, 4478 were excluded for not having both a systolic and diastolic phase in the archive. Of the remaining 1495 scans, 1357 were excluded for not having qualifying solid PNs in both systolic and diastolic phases, and 31 scans were excluded after consensus decision (as not representing true nodules).

From the 107 scans included, a total of 195 nodules were identified with a manual long-axis measurement between 5 and 8 mm. The mean age of the patients was 67.8 years, with a male to female ratio of 1.34 (Table 1). Nodules were more frequently identified in the upper thirds of the FOV in the coronal plane (49.2%) and between the middle (39.5%) and posterior (40.5%) thirds in the axial plane.

**Table 1:** Demographics of the sample population.

		(n = 195)
Age (yrs): M ± SD		67.8 ± 11.7
Gender: n (%)	Male	112 (57.4)
	Female	83 (42.6)

M = Mean, SD = standard deviation

**Figure 2.**
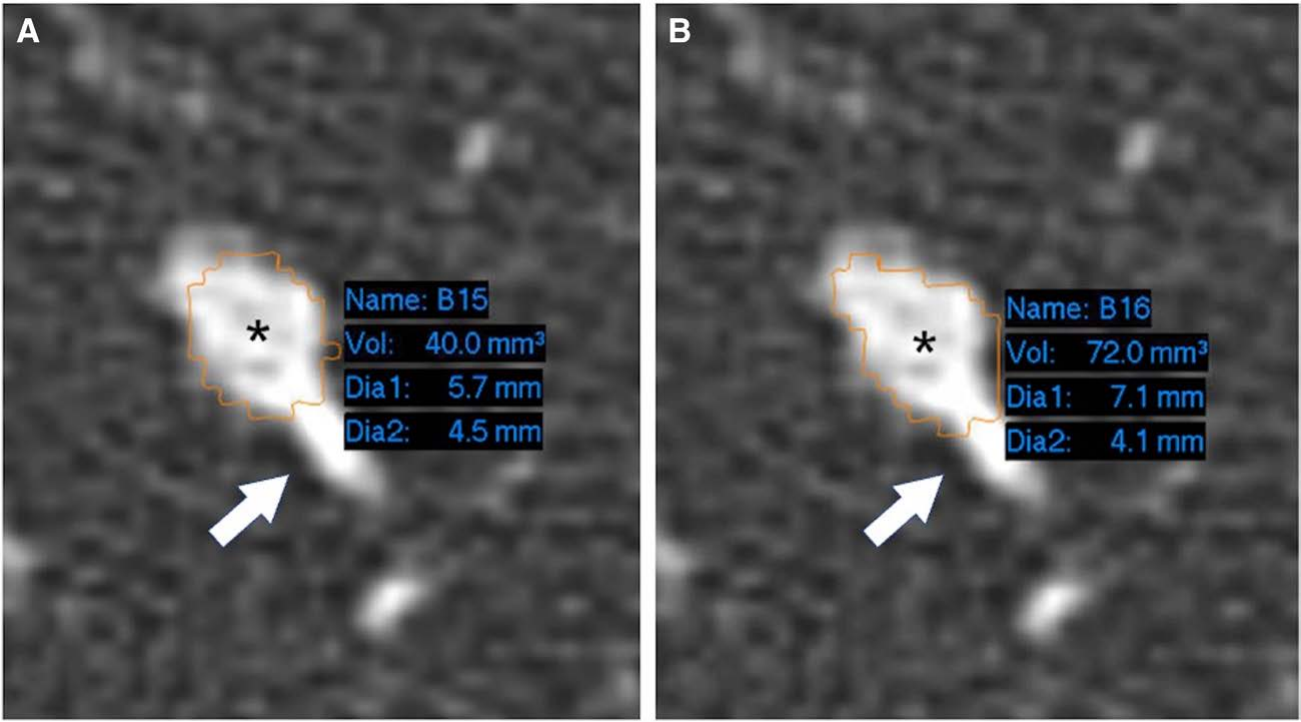
Example of a nodule included in the study measured in systole (A) and diastole (B). The seed points (*) were placed in the nodule center, and the yellow lines show the semiautomatic segmentation result, which was not corrected and considered adequate by both observers. Notice the small vessel (arrows) approaching the nodule, which was excluded from the segmentation in both instances. The volume measured in diastole was 80% greater than in systole.

For each nodule, each set of volume measurements was repeated by each observer, for systole and diastole, and using each software package, resulting in a total of 1560 volume measurements (8 measurements per nodule). Figure 2 provides an example of measurement. The quality of the nodule segmentation was considered inadequate in more cases using 1 software package (tool 1; n = 157; 20.1%) than the other (tool 2; n = 95; 12.2%).

The mean volume was close to 50mm^3^ with a median of 35mm^3^, ranging from 10.4 to 400 mm^3^ (Table 2).

**Table 2:** Descriptive statistical analysis of volume measurements in the sample population and at different phases of the cardiac cycle, using different software packages and by different observers.

Volume	n	Mean (mm^3^)	SD (mm^3^)	Q_1_ (mm^3^)	Median (mm^3^)	Q_3_ (mm^3^)
Sample population						
	1237	63.282	68.039	24.5	38	69
Between systole and diastole						
Systole	645	51.99	46.11	23.375	36	62
Diastole	645	50.836	44.947	23.3	35.5	60.4
Difference	645	−1.154	13.13	−3.6	−0.5	1.7
Between different software packages						
Tool 1	616	47.108	38.438	23.3	34.5	59.8
Tool 2	616	48.807	42.257	22	35	59
Difference	616	1.699	15.942	−3.25	−2.0	6.7
Between different observers						
Observer 1	642	53.62	46.993	24	36.6	62.9
Observer 2	642	52.91	47.172	23.4	36.2	62
Difference	642	−0.701	8.723	0	0	0
Between different observers (systolic dataset)						
Observer 1	318	44.668	29.936	23.4	34.9	59
Observer 2	318	43.751	29.583	23	34.05	58
Difference	318	−0.917	9.911	0	0	0
Between different observers (diastolic dataset)						
Observer 1	322	43.862	29.316	23	34.6	57.2
Observer 2	322	43.019	28.611	23	34	55
Difference	322	−0.842	6.806	0	0	0

Q_1_ = first quartile, Q_3_ = third quartile, SD = standard deviation.

The volume measurements of the solid PN show a very strong association between systole and diastole, with a Kendall tau correlation coefficient (τ) of 0.812 (CI_95_ = [0.785; 0.838], df = 645, *P* < .0001); between software packages with a τ = 0.744 (CI_95_ = [0.714; 0.771], df = 616, *P* < .0001); and between different observers, with a τ = 0.942 (CI_95_ = [0.916; 0.957], df = 682, *P* < .0001) globally; τ = 0.928 (CI_95_ = [0.874; 0.955], df = 338, *P* < .0001) for the systolic dataset; and τ = 0.956 (CI_95_ = [0.931; 0.971], df = 344, *P* < .0001) for the diastolic dataset.

**Figure 3.**
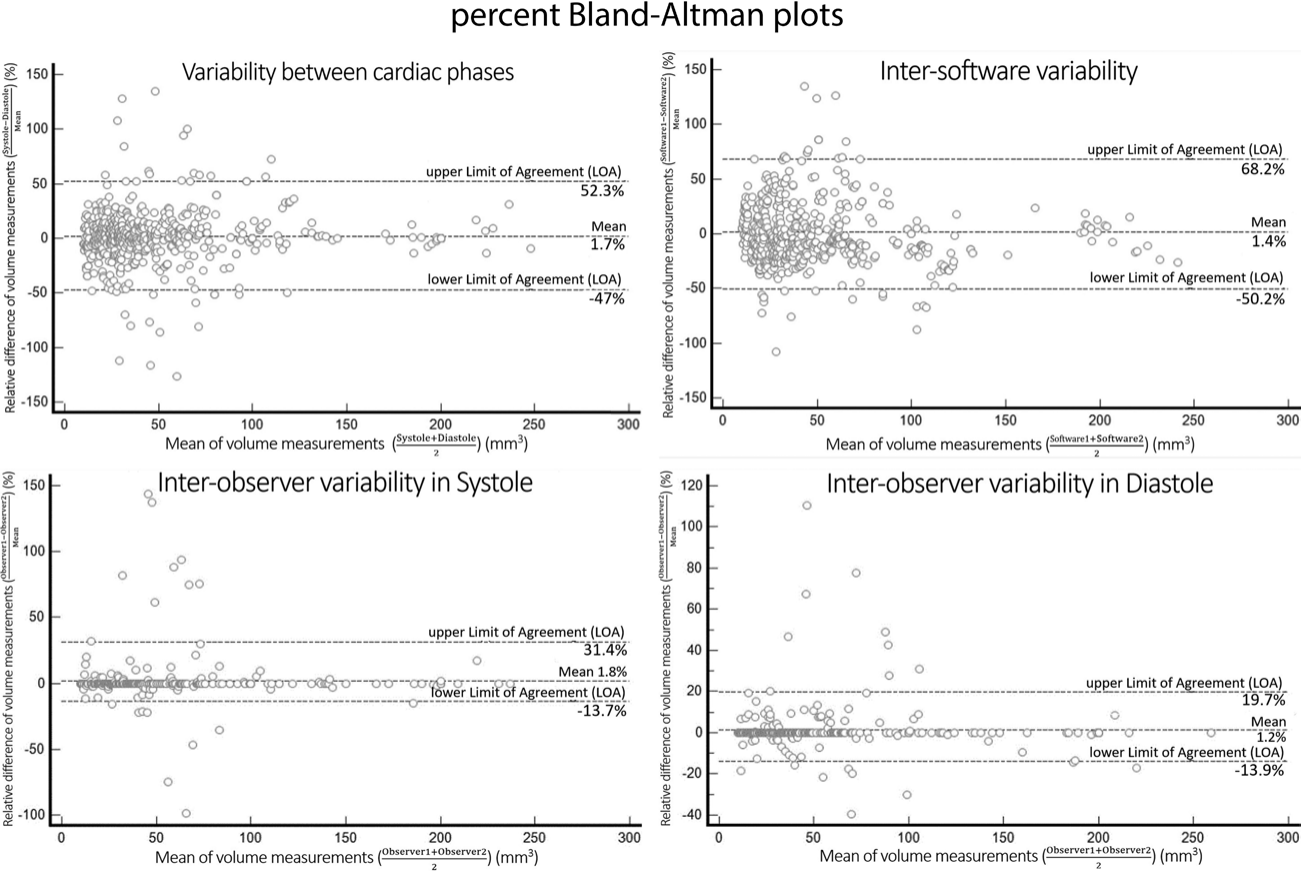
Relative (percent) Bland-Altman plots, with estimated limits of agreement, when comparing volume measurements between systole and diastole (top left corner), different software packages (top right corner), and different observers (bottom). Interobserver subanalysis is presented for the systolic (bottom left corner) and diastolic datasets separately (bottom right corner).

The Bland-Altman plots show an increase in the differences’ variability as the measurement’s magnitude increases, particularly for different cardiac phases and software packages. The estimated LOA of the percent Bland-Altman plots are [−47.0%; 52.3%], between phases of the cardiac cycle, and [−50.1%; 68.2%] between software packages (Fig. 3 and Table 3), with no significant bias. The estimated LOA of the percent Bland-Altman plots between different observers are [−14.5%; 27.8%] globally, [−13.6%; 31.4%] for the systolic dataset and [−13.9%; 19.7%] for the diastolic dataset.

**Table 3:** Results of the Bland-Altman analysis.

	Bland-Altman (mm^3^)			Percent Bland-Altman (%)		
	Bias	lower LOA	Upper LOA	Bias	lower LOA	Upper LOA
Between systole and diastole	1.15	−24.91	36.76	1.73	−47.02	52.29
Between software packages	1.7	−39	34.08	1.35	−50.16	68.21
Between observers	0.7	−6.2	17.39	1.49	−14.45	27.77
Between observers (systolic dataset)	0.88	−4.7	24.47	1.75	−13.63	31.36
Between observers (diastolic dataset)	0.53	−8.58	15.85	1.23	−13.92	19.66

LOA = estimated limits of agreement.

## 4. Discussion

Growth estimation is feasible using PN volumetry on the diastolic phase of CCTA scans. Conversely, comparing volume measurements from different cardiac phases could be as unreliable as using different volumetry tools (similar measurement variability), implying a lower discriminating power between benign and malignant pathology.

For the Bland-Altman analyses, we defined the a priori LOA as a difference of ±25% in volume, according to the previously reported variability of PN volumetry in LD-CT scans, which form the basis of recommendations for waiting time between follow-up scans.^[15,23,28]^

In the study’s results, the shapes of the Bland-Altman plots for different software tools and cardiac phases show that the difference in volume increases proportionally with their mean.^[27]^ Therefore, the variability is assumed to be relative or proportional (represented as a volume percentage). However, this proportionality is less obvious between different observers with larger nodules. For example, for PNs larger than 65.6 mm^3^, the estimated LOA between observers vary between −8.6 mm^3^ and 15.9 mm^3^, which will be <25% of their volume. The proportionality seems to apply for smaller nodules, so the percentage Bland-Altman analysis is still relevant with the estimated LOA ([−14.45%; 27.77%]) much closer but outside the a priori LOA. However, in the inter-observer subanalysis specific for diastolic measurements, the estimated LOA ([−13.92%; 19.66%]) are well within the a priori LOA. The lower variability seen in diastole is likely related to less cardiac motion when compared with systole and the higher temporal resolution compared to LD-CT scans. In addition, the low interobserver variability is an expected benefit of semiautomatic volumetry tools.^[15]^

Identified limitations of the present study include (1) the large portion of excluded candidate CCTA scans, which was due to departmental protocol not to archive, per default, both systolic and diastolic phases; (2) the disproportionate representation of smaller nodules in comparison to larger ones; and (3) the non-normal distribution of the differences between measurements, which implied a nonparametric statistical approach.

To the best of our knowledge, this is only the second (and largest) study to focus on PN volumetry in ECG-gated CT scans after Boll et al suggested that factors related to the pulmonary circulation (cardiac phase and cardiac motion) could influence nodule segmentation.^[21]^ It is also the first study to measure the in vivo variability of PN volumetry on CCTA, which is the main requirement for growth estimation. Therefore, our conclusions still need to be reproduced and validated independently, and further research is needed with a larger sample size before volumetry of PN in CCTA scans becomes useful for growth estimation between 2 CCTA scans. Further research should also compare in vivo volume measurements between CCTA in diastole and LD-CT, before growth of PN can be estimated by comparing CCTA and LD-CT scans (as used in Lung cancer screening), since significant protocol differences exist.

The increased variability of volume measurements between cardiac phases could model the effect of hemodynamic changes often seen between follow-up scans, like the acute onset of heart failure or pleural effusion. Such events are likely more significant than the relatively small intrascan changes seen between systole and diastole. Since the actual in vivo volume of a PN is unknown, a common approach in studies on PN volumetry is to use “coffee-break” studies, where the PN is scanned twice in a short time frame (typically minutes), thus excluding any real growth. In this way, measurement variability explains any volume difference.^[15,26,27]^ However, the impact of such hemodynamically significant events is difficult to study directly since the absence of growth cannot be assumed over any substantial period. In addition, the extra radiation and time demands of “coffee-break” studies make them more suited to phantom studies. On the other hand, CCTA scans allow several opportunities to measure the volume of a PN at different cardiac phases and within a single scan (i.e., no growth occurred), thus providing a tool better suited for clinical research.

## 5. Conclusions

The interobserver variability of PN volumetry on CCTA scans was comparable to the reported for LD-CT only in diastole. As such, growth estimation of PN between 2 CCTA scans could be feasible using diastolic measurements. However, using systolic volume measurements or comparing volume measurements from systole and diastole implies a higher variability, which could lower the discriminating power of PN volumetry.

## Acknowledgments

We would like to acknowledge Prof Paulo Pereira for statistical support.
